# Implementing emergency admission risk prediction in general practice: a qualitative study

**DOI:** 10.3399/BJGP.2021.0146

**Published:** 2021-11-16

**Authors:** Bridie Angela Evans, Jeremy Dale, Jan Davies, Hayley Hutchings, Mark Kingston, Alison Porter, Ian Russell, Victoria Williams, Helen Snooks

**Affiliations:** Swansea University Medical School, Swansea, Wales.; Warwick Medical School, University of Warwick, Coventry.; Swansea University Medical School, Swansea, Wales.; Swansea University Medical School, Swansea, Wales.; Swansea University Medical School, Swansea, Wales.; Swansea University Medical School, Swansea, Wales.; Swansea University Medical School, Swansea, Wales.; Swansea University Medical School, Swansea, Wales.; Swansea University Medical School, Swansea, Wales.

**Keywords:** qualitative research, emergency service, hospital, general practice, health risk appraisal, health service evaluation, chronic disease

## Abstract

**Background:**

Using computer software in general practice to predict patient risk of emergency hospital admission has been widely advocated, despite limited evidence about effects. In a trial evaluating the introduction of a Predictive Risk Stratification Model (PRISM), statistically significant increases in emergency hospital admissions and use of other NHS services were reported without evidence of benefits to patients or the NHS.

**Aim:**

To explore GPs’ and practice managers’ experiences of incorporating PRISM into routine practice.

**Design and setting:**

Semi-structured interviews were carried out with GPs and practice managers in 18 practices in rural, urban, and suburban areas of south Wales.

**Method:**

Interviews (30–90 min) were conducted at 3–6 months after gaining PRISM access, and ∼18 months later. Data were analysed thematically using Normalisation Process Theory.

**Results:**

Responders (*n* = 22) reported that the decision to use PRISM was based mainly on fulfilling Quality and Outcomes Framework incentives. Most applied it to <0.5% practice patients over a few weeks. Using PRISM entailed undertaking technical tasks, sharing information in practice meetings, and making small-scale changes to patient care. Use was inhibited by the model not being integrated with practice systems. Most participants doubted any large-scale impact, but did cite examples of the impact on individual patient care and reported increased awareness of patients at high risk of emergency admission to hospital.

**Conclusion:**

Qualitative results suggest mixed views of predictive risk stratification in general practice and raised awareness of highest-risk patients potentially affecting rates of unplanned hospital attendance and admissions. To inform future policy, decision makers need more information about implementation and effects of emergency admission risk stratification tools in primary and community settings.

## INTRODUCTION

Using computer software in primary care to predict risk of emergency hospital admission is widely advocated to support the proactive care of patients who are vulnerable and to manage demand on healthcare services.^1^^–^4 In 2012–2013, there were 5.3 million emergency admissions to hospitals in England, costing £12.5 billion;^5^ approximately half of these came from 5% of the population, yet an estimated one in five is avoidable.^6^^–^8

Using predictive risk stratification in general practice allows clinicians to identify patients at high risk of emergency admission,^8^^–^14 and target care and services according to that level of risk.^14^^–^16 Individual risk scores are estimated based on past use of health care, diagnoses, and medications, and tools are generally more accurate and consistent than clinical opinion.^17^ The rationale is that the targeted management of patients can reduce emergency admissions to hospital, improve patient outcomes and experience, and provide better value for money;^14^^,^^16^ however, there is little evidence to support this. A 2015 systematic review and meta-analysis of 36 studies showed no significant differences in total cost, mortality, or utilisation of primary or secondary care when case management was used to support patients who were vulnerable;^18^ in addition, a 2013 review of six stratification tools was criticised for misleading presentation of findings.^19^ Even so, predictive risk stratification is widely promoted in policy, both internationally and across the UK,^11^^–^13^,^^20^^–^22 with recent incentive schemes focusing on patients with the highest level of risk.^23^^,^^24^

Implementation of the Predictive Risk Stratification Model (PRISM) emergency admissions risk stratification tool in Wales was evaluated.^25^ The trial — Predictive RIsk Stratification Model: A Trial In primary Care (PRISMATIC) — was a randomised, stepped-wedge trial with a qualitative component,^10^^,^^26^ which found that, contrary to expectations, the predictive risk stratification increased emergency admissions to hospital; full results are reported elsewhere.^27^ Here, the qualitative work exploring the implementation of PRISM in general practice is reported; this study aimed to explore the views and experiences of GPs and practice managers who used the PRISM risk stratification tool.

## METHOD

### Theoretical framework

Implementing new healthcare technologies can be slow and difficult,^28^^,^^29^ and Normalisation Process Theory (NPT)^30^ is increasingly used as a conceptual framework to examine and explain this.^31^ NPT considers implementation as a process, entailing sustained work by those responsible, and suggests four constructs that help individuals to understand how innovation occurs in routine practice:

- coherence — how people understand the innovation and its purpose;
- cognitive participation — what decisions are taken to use it, based on perceived advantages;
- collective action — what people do to bring the innovation into everyday use; and
- reflexive monitoring — how an innovation is reviewed, modified, or abandoned.^32^

**Table:** How this fits in

UK policy has incentivised the use of risk prediction stratification in primary care to reduce emergency hospital admissions, despite a lack of evidence about process or effect. In a trial evaluating the Predictive Risk Stratification Model (PRISM) in general practice, increased emergency and hospital admissions were reported. To understand implementation, interviews were conducted with GPs and practice managers who reported using PRISM on a small group of patients at high risk of emergency admission to hospital. Although the interviewees doubted there was any impact on care, they reported PRISM raised their awareness of patients in the highest-risk groups, which might affect unplanned hospital attendance and admissions. Raised awareness of these issues could influence GPs likelihood of seeking emergency hospital care for a patient, or it could make patients more aware of their health needs leading them to seek emergency hospital care.

The authors have previously reported on coherence, the first of these constructs, which had been explored in focus groups with GPs and other practice staff before PRISM was introduced.^33^ Staff welcomed the opportunity to use the tool, with some dubbing it a ‘golden goose’ for its potential to both benefit patients and manage demand on health services.^33^ In the study presented here, the other three constructs of NPT — cognitive participation, collective action, and reflexive monitoring — are used to shape the analysis of the experiences and the reflections of GPs and practice managers, after they received access to PRISM.

### Design and setting

PRISMATIC took place in 32 practices in south Wales. The stepped-wedge design (also known as randomised multiple interrupted time-series or progressive cluster randomised trial)^34^ enabled all participating practices to implement PRISM during the 18-month study. All practices began as control practices without PRISM, then each month, over the course of a year, two or three practices received training and access to the tool. As the trial progressed, the number of intervention practices increased and the number of control practices fell. This design aimed to protect against some sources of bias, including inherent differences and contamination between practices, as well as the resentful demoralisation of controls unable to use the intervention.^35^

During the study period, the Welsh Government introduced a financial incentive, through the Quality and Outcomes Framework (QOF), to encourage GPs to use emergency admission risk stratification tools to support hospital avoidance^24^ (see Supplementary Box S1). QOF tasks to support hospital avoidance included:

- producing a list of 5% of patients in the practice predicted to be at increased risk of unscheduled admission;
- reviewing 10% of patients on that list (maximum 0.5% of practice list) and preparing an active management plan; and
- holding at least four meetings a year to review care for those patients identified.

The local Health Board encouraged practices to use PRISM for this work and payment was made when the completed QOF tasks were submitted to the Health Board. The phased roll-out meant practices had access to PRISM for different lengths of time during this period. An overview of PRISM is given in Box 1. All participating practices were remunerated (up to £1250) for supporting the study, including participating in interviews.

**Box 1.** The PRISM risk stratification tool.

**Overview** PRISM generates a predicted risk score (out of 100%) of emergency admission to hospital for each patient on a practice list that is updated monthly. It stratifies: the general practice population into four levels, based on the individual patient’s risk of an emergency admission to hospital in the following 12 months; and patients into four risk groups according to the relative risk within the practice as a whole, regardless of individual characteristics and comorbidities (default stratification). As an example, using the default stratification, the 0.5% of patients at highest practice risk will appear in level 4; those in the next 4.5% in level 3 (moderate risk); those in the next 15% in level 2 (low risk), and the remaining 80% in level 1 (very low risk).^36^ The variables used to develop PRISM were drawn from routinely available data on inpatient, outpatient, and primary care episodes, and from the Welsh Index of Multiple Deprivation, which includes data on employment, income, housing, environment, education, and health. **Support and training** GPs were invited to a practice-based training session on using PRISM to identify patients at risk of emergency admission. A user-friendly handbook was provided, along with access to clinical support through two locally appointed GP champions; technical support was delivered via email or telephone to the Primary Care Service Desk of NHS Wales Informatics Service. Individual practices were advised that they could choose how to use the tool in their practice — for example, how often they interrogated the data; whether they reviewed all patients or subgroups, such as risk levels or diagnosed conditions; whether they accessed a patient’s risk score during a consultation; and what action they took based on a patient’s risk score. Each practice nominated a GP lead, who was responsible for coordinating the use of PRISM and the participation in PRISMATIC, including engagement with other clinical and practice staff.

*PRISM = Predictive Risk Stratification Model. PRISMATIC = Predictive RIsk Stratification Model: A Trial In primary Care.*

### Data collection

GPs and practice managers from 18/32 participating practices were purposively sampled to cover a range of practice sizes, geographic settings, and socioeconomic spread. The lead GP or practice manager in each practice was interviewed at two time points:

- 3–6 months after PRISM was activated in their practice (June 2013–July 2014);
- at the end of the intervention phase, up to approximately 18 months after it was available in the first practices (June–December 2014).

Two experienced researchers from the study team conducted all interviews, which were held at general practices and lasted 30–90 min. During interviews, responders were asked to describe how they used the tool, and probes were used to explore their comments. This enabled the researchers to understand how the tool was introduced and implemented, and ascertain changes over time. With participants’ consent, interviews were recorded; they were then independently transcribed, with all personal and geographic identifiers removed. Field notes were made after each interview. Interview schedules are detailed in Supplementary Boxes S2 and S3.

### Analysis

Interview transcripts were analysed thematically, informed by NPT as the underlying theoretical framework. Thematic analysis is a systematic and transparent method that generates themes from the explicit and implicit ideas in the original accounts of participants.^37^^,^^38^ Team members — the two interviewers, two other researchers, and a service user — read the transcripts and developed a coding framework informed by the NPT framework. One of the interviewers led the analysis; the other researchers independently supported key stages of coding, taking into consideration consistency or deviation of views across the sample, and generating themes and interpretation, thereby encouraging a critical stance to test and confirm the findings.^39^^,^^40^

### Service-user involvement

Service-user involvement has been reported according to the Guidance for Reporting Involvement of Patients and the Public (GRIPP).^41^ Two service users were recruited who, throughout the study, were collaborators in the research partnership.^42^^–^44 As members of the research management group, they were responsible for strategic and operational decisions, and contributed as equal team members to ensure patients’ perspectives were considered at all stages of the study. One of these service users was also involved in the data analysis.

Both service users were recruited through Service Users with Chronic Conditions Encouraging Sensible Solutions (SUCCESS), a group of patients and carers engaged in research linked to chronic conditions management policy in Wales. They reported regularly to SUCCESS and sought feedback to inform their contributions to PRISMATIC.^45^ Two other service users were recruited to the trial steering committee through Involving People^46^ to ensure their independence; in addition, best practice was followed by the researchers, ensuring all users received honoraria, expenses, training and support, a named contact, information, and networking opportunities.^47^

## RESULTS

At timepoint 1, all practices contacted for interview responded (a 100% response rate). At timepoint 2, one GP had left their practice and the practice manager was not available (a 95% response rate).

In total, 22 interviews (GPs, *n* = 18; practice managers, *n* = 4; practices, *n* = 18) were conducted at timepoint 1, and 19 interviews (GPs, *n* = 17; practice managers, *n* = 2; 17/18 practices were contacted for interview) at timepoint 2. One GP left their practice between the first and second interviews, and two practice managers were not available at the second interview. Results are presented for three constructs of NPT — cognitive participation, collective action, and reflexive monitoring — having covered coherence elsewhere.^32^ Quotations are illustrative and typical of responders’ comments, unless otherwise stated.

### Cognitive participation: deciding to use PRISM

Responders described how they made decisions about using PRISM based on its perceived advantages. There was a consistent message that it was used, in the main, to identify patients at high risk of emergency admission to hospital to fulfil QOF requirements:

*‘It was fantastic because we were able to pick out the patients that the local health board had highlighted for the QOF thing.’*

(GP11, interview 2)

*‘... we only really wanted to know what we needed to know to do the piece of work that we were gonna get judged on.’*

(GP32, interview 1)

A few practices reviewed and refreshed their PRISM list throughout the trial period. Responders said the majority of patients were known and considered to be at high risk, but some names were unexpected or unfamiliar. During the second interview at the end of the study, only two practices reported that they were still using PRISM after completing and submitting their QOF reports to the local Health Board.

A few GPs, who had access to PRISM earlier in the study reported using it to support patient care in ways outside of QOF requirements; they interrogated the PRISM data to understand the reason for a high score or to review specific patient groups, such as patients with chronic obstructive pulmonary disease. As GP13 stated in the second interview:

*‘* [We have been] *Looking at patients that’ve been deemed high risk, but not necessarily the highest risk, people that we might be able to do something about. Just exploring the data and seeing if there is anything that we could do to be more proactive.’*

### Collective action: what GPs and practice managers did to bring PRISM into use

Bringing PRISM into use involved three processes:

- using the technology itself;
- sharing the information it generated with relevant clinical teams; and
- taking action with patients in light of the information generated.

In their first interview, GP6 noted:

*‘Within the first couple of weeks of having it* […] *we all sat down and we went through the ones who were on the top … what we thought about it and what interventions we thought might work — without any plan, we just discussed it.’*

Typically, the lead GP or practice manager generated a list of patients in the top stratum of risk and printed this or saved the information in a spreadsheet. A few practices also created a screen pop-up on the record of those patients at high risk, which alerted all staff to tailor care or make early appointments when these patients telephoned reception. As GP6 noted in their second interview:

*‘That flags up — that’s like a warning sign that maybe we should take them a bit more seriously.’*

PRISM was not without technical challenges, such as slow broadband speeds, system crashes, and passwords locking. Some responders also complained that the tool was not integrated with practicebased clinical information, which inhibited the routine management of patient data:

*‘What I wanted was to download my 53 patients … the information that would allow me to work out why they’re on that list … and it really disappointed me that a lot of that I had to do manually.’*

(GP15, interview 1)

Responders described sharing the list of individual patients’ risk scores at routine practice meetings for discussion when time allowed or, if the QOF deadline was close, dividing it among partners who worked individually.

More than half of responders said the work of bringing PRISM into use was inhibited by:

- the many demands on GPs’ time;
- shortage of GPs due to illness;
- retirement; or
- maternity leave.

GPs and practice managers reported a range of actions after using PRISM. These generally constituted small amendments to supplement existing care for individual patients or extra reviews (some through house calls) to fine-tune current treatment:

*‘There will have been people who were reviewed or assessed, who otherwise wouldn’t have been.’*

(GP14, interview 2)

Some reviews explicitly focused on how best to manage crises that might lead to emergency admission, with one GP reporting what they had said to a patient during a face-to-face consultation:

*‘“Look, you’ve been admitted on a number of occasions. Obviously, the chances of you being admitted are quite high; why don’t we do something a bit different? We will try and alter your medication to maybe control your condition a bit better. We are here during the day so use us, rather than dial 999, and we can get somebody to see you”.’*

(GP11, interview 2)

Other effects on patient care were reported. One GP described a nurse talking to patients at high risk of emergency admission about identifying infections, managing weight, and spotting early warning signs. In their second interview, GP19 stated:

*‘We got these patients and tried to* [take] *more time in educating them … you teach them what to do.’*

(GP19, interview 2)

In another practice, emergency drug packs were made up for identified patients to use at weekends and bank holidays if their health deteriorated. Some high-risk patients were referred to outpatient clinics, nursing teams, or other care agencies for non-medical needs.

All responders reported difficulty changing from a reactive, to a proactive, approach to care when the daily routine in general practice was so busy. Some also said they were concerned that practice staff did not have the capacity or skills to take on more patients.

### Reflexive monitoring: reviewing PRISM

GPs and practice managers generally judged it unlikely that PRISM had any effect on emergency admissions and emergency department (ED) attendances; there was a widespread feeling that admissions initiated by GPs were already low, with little scope for further reductions:

*‘There are odd occasions where you were able to proactively help somebody or put a plan in place to stop them being admitted to hospital. I think it’s a fallacy to think that you could reduce emergency admissions from primary care because the primary care admissions are so small ... one case a week, if that.’*

(GP02, interview 2)

A minority of responders could identify instances in which an emergency admission may have been avoided and two GPs, who targeted patients with frequent ED attendance, reported that those patients’ use of emergency services had fallen.

However, one GP suggested how PRISM may have been associated with increased hospital admissions:

*‘We did bring in a certain number of people and do care plans with them, and then we ended up admitting them because we’d seen them and they looked unwell.’*

(GP31, interview 2)

The majority of responders described how PRISM changed their awareness of those patients at high risk of emergency admission to hospital, especially when patients unexpectedly appeared in the top stratum:

*‘It* [a high risk score] *does have an effect of making you sit up and think “heck, what’s he doing up?”’*

(GP05, interview 2)

One practice manager observed changes in GPs’ attitudes and behaviours towards identified high-risk patients:

*‘Made some clinicians aware of them* [high-risk patients] *, initiated home visits, changes to medications, getting other people involved in their care.’*

(practice manager [PM]02, interview 2)

GPs said the combination of PRISM and the QOF incentive increased their contact with some patients in this high-risk group so they could reassure themselves that these patients were receiving optimum care:

*‘We’ve probably had more talks together, as a group, as to how we have contacted the ambulance service and A&E* [accident and emergency] *... And we’ve talked about these patients more I think.’*

(GP18, interview 2)

Most believed that the increased GP–patient interaction was probably beneficial, regardless of any treatment delivered, because patients appeared to appreciate the extra attention and advice; however, being careful not to alarm patients and precipitate self-referrals, GPs did not generally tell them about their risk score.

Some responders felt that the QOF’s focus on patients at highest risk was misplaced, as these were already well known to the practice. They believed that patients at medium risk of emergency admission had most to gain from close attention and proactive care, if resources were available:

*‘Those in the middle bracket were the kind of patients that you were possibly able to help more than those ... in the higher echelons that were already having all the input that was available … because you can actually put in things that will stop them from going up the pyramid.’*

(GP02, interview 2)

*‘I think QOF work highlighted our lack of support in managing these individuals. So, although they did have medical case review, that didn’t really generate much extra activity, particularly almost no response from district nursing.’*

(GP08, interview 2)

Responders reflected that the QOF payments encouraged short-term use of PRISM, in the absence of extra resources to support changing practice in the long term. Although a small number of responders reported referrals to non-medical services, the majority suggested that the provision of community health and social services was inadequate to support proactive care for the patients they reviewed who were at high risk of emergency admission to hospital:

*‘We discussed those patients at various meetings; we made plans about how to minimise admissions, access out of hours, casualty, but it’s not in this year’s QOF … it was more a question of a useful tool to achieve points, more than anything else.’*

(GP10, interview 2)

## DISCUSSION

### Summary

This study identified a range of, often contrasting, views about the use and usefulness of PRISM in general practices. GPs and practice managers reported that the decision to use PRISM was based mainly on fulfilling QOF requirements. In addition, it was generally applied for a short period to a very small number of patients who were at high risk of emergency admission; only a minority used PRISM in other ways, such as identifying patients at medium risk. Although most responders were well aware of who their high-risk patients were, GPs said their awareness of these individuals was heightened by knowing the PRISM scores.

Bringing PRISM into practice was inhibited by it not being integrated with practice systems, and information sharing was generally done in practice meetings. Systemic barriers — such as other demands on GPs’ time, a shortage of GPs, and software and technical problems — seemed to be temporarily overcome by the QOF incentive to use the tool.

Changes to the care of high-risk patients, as a result of using PRISM, were diverse and generally small in scale, such as extra visits, care plan reviews, medication amendments, tailored self-care advice, and referrals to other services.

Responders’ evaluation of PRISM was mixed: there were doubts about it having any large-scale effect, but many cited effects likely to have benefited individual patients. Some concerns were expressed about how the QOF influenced the use of PRISM; in particular, the focus on patients at the highest risk of emergency admission, who may have been least suitable for proactive management, and the short-term nature of the implementation were noted.

### Strengths and limitations

Interviewees comprised GPs and practice managers with a wide range of experiences across 18 diverse practices in different locations across Wales. The authors purposively sampled practices of different sizes and caring for patients in areas with varied socioeconomic and urban/rural characteristics, to be typical of general practice in Wales.

All 18 practices contacted took part in the interviews, which, conducted at two periods, provided rich data on implementation and change over time. Consistency of experience across diverse responders in varied settings suggests the findings are generalisable. These practices volunteered to take part in PRISMATIC, committing themselves to using the PRISM tool for a fixed time and receiving a small payment. It is important to be aware that their views may not be typical of the views of the practices that did not take part in the study.

The researchers examined participation, action, and reflections among GPs and practice managers who used PRISM, however, the views of local commissioners and health service managers are not known.

### Comparison with existing literature

Software in general practices that predicts risk of emergency hospital admission for every registered patient has been widely promoted as a means of targeting preventive services to people at high risk to avoid crises that result in emergency admissions.^10^^,^^23^ However, main PRISMATIC findings showed that the introduction of PRISM resulted in a statistically significant increase in emergency hospital admissions and use of other NHS services, with no evidence of benefits to patients or the NHS.^27^

One interpretation is that the increases arose from changed awareness and behaviour among GPs and other practice staff, particularly when individuals in the top stratum were not expected to be there; this made practitioners more cautious in their clinical practice, leading to overdiagnosis that may, in turn, lead to emergency admissions. It is also possible that patients (and their carers) who received extra contact became more aware of their poor health and sought emergency care when they previously would not have done.^48^^–^51 Additionally, it may be that GPs had less time for other patients, leading to them needing emergency hospital care.^52^

GPs and practice managers in these interviews were restrained in their assessment of PRISM and uncertain of any clear consequences for patients or health services. They had few options for enhancing care to patients identified as being at high risk because of very limited access to community services, which is known to be important when targeting such individuals and reducing emergency admissions.^53^^,^^54^

The PRISMATIC trial was highly powered — the inclusion of >200 000 people registered to the participating general practices and >1000 at the highest level of risk meant that it involved enough participants to be able to detect small differences in outcomes. Consequently, moderate behaviour changes at individual surgeries contributed to statistically significant increases in NHS activity across the 32 participating practices. The qualitative findings presented here suggest small behaviour changes, which practice staff may barely have noticed.

It should also be acknowledged that the QOF requirements may have affected GP behaviour and opportunities for innovative working. The QOF incentive scheme, in which specific issues are targeted using short-term payments, has been associated with adding an administrative and time-consuming load to practice work and limiting multidisciplinary activity to coordinate care for patients with multiple needs.^55^

The responders in this study acknowledged that QOF was the context within which they initially used, and subsequently did not re-use, PRISM; it is possible that the potential benefits of incorporating risk-prediction software into general practice were unrealised because of the way the incentive scheme was structured.

### Implications for research

The primary hypothesis of the PRISM intervention is that identifying people at high risk of emergency admission to hospital can facilitate further targeted care, thereby reducing those admissions. This assumption appears to underlie wide implementation of predictive risk stratification tools in primary care across the UK, without evidence that expected reductions in emergency admissions would actually be achieved.^18^^,^^26^^,^^27^^,^^56^

Logic suggests such software should align with other policy-promoted interventions to improve patient health and wellbeing, such as integrated care, case management, and a focus on the socioeconomic and lifestyle factors that affect quality of life.^56^^–^59 However, findings from the PRISMATIC trial illustrate the unpredictable consequences of introducing service innovations into NHS practice.

The qualitative data presented here do not entirely explain the quantitative findings of a rise in NHS activity when the risk prediction tool was used in general practices;^27^ however, they do offer insight into the changed perceptions and behaviours of general practice staff after high-risk patients were identified by the tool and they were incentivised to review care for these patients. In this case, it could be that the intervention identified new unmet need or that, in the context of a focus on risk, clinicians lowered their threshold for admission. These likely changed approaches help to understand the unexpected trial results.

The results also highlight the extent of the complexity of adopting and using innovation in the real world, and how seemingly beneficial incentive schemes may distort outcomes

It is not uncommon for implementation to proceed before supporting research evidence is available;^60^ assumptions are made about mechanisms that do not necessarily reflect reality. Embedding this qualitative work in the PRISMATIC trial responded to calls for a thorough understanding of how new services are adopted and used because organisational culture affects implementation.^61^^,^^62^

The findings presented here highlight the need for further research into behaviours and attitudes in general practice to inform use of emergency admissions risk stratification tools as they are available.^56^ The researchers plan to carry out further research to explore these potential mechanisms of change.

To conclude, emergency admission risk stratification tools are widely advocated to reduce emergency hospital admissions and are available in primary and community care across much of the UK. However, there is a lack of evidence to support the view that they enable proactive care and improve patient outcomes.

This study revealed varied views and experiences among GPs and practice managers about use of the PRISM tool, which was short term and driven by external factors, rather than embedded in new ways of working.

Raised awareness of patient risk and focusing attention on the small numbers of patients at greatest risk may explain quantitative trial findings of increased emergency hospital admissions and use of other NHS services. Decision makers need more information about the implementation and effects — both positive and negative — of such emergency admissions risk stratification tools in primary and community settings to inform future policy on their use. There is now uncertainty about how to use the widely available predictive risk tools in order to achieve intended effects.

The authors recommend further research about costs, effects, mechanisms of change, and patients’ views on using emergency admission risk prediction tools in primary care in order to inform policy and practice.
